# Activation of adenosine A_3_ receptor protects retinal ganglion cells from degeneration induced by ocular hypertension

**DOI:** 10.1038/s41419-020-2593-y

**Published:** 2020-05-27

**Authors:** Raquel Boia, Manuel Salinas-Navarro, Alejandro Gallego-Ortega, Caridad Galindo-Romero, Inês D. Aires, Marta Agudo-Barriuso, António Francisco Ambrósio, Manuel Vidal-Sanz, Ana Raquel Santiago

**Affiliations:** 1 University of Coimbra, Coimbra Institute for Clinical and Biomedical Research (iCBR), Faculty of Medicine, Coimbra, Portugal; 2 University of Coimbra, Center for Innovative Biomedicine and Biotechnology (CIBB), Coimbra, Portugal; 3 Clinical Academic Center of Coimbra (CACC), Coimbra, Portugal; 4 Instituto Murciano de Investigación Biosanitaria-Virgen de la Arrixaca (IMIB-Arrixaca), Murcia, Spain; 5 Departamento de Oftalmología, Facultad de Medicina, Universidad de Murcia, Murcia, Spain; 6 Association for Innovation and Biomedical Research on Light and Image, Coimbra, Portugal

**Keywords:** Diseases, Preclinical research

## Abstract

Glaucoma is a progressive chronic retinal degenerative disease and a leading cause of global irreversible blindness. This disease is characterized by optic nerve damage and retinal ganglion cell (RGC) death. The current treatments available target the lowering of intraocular pressure (IOP), the main risk factor for disease onset and development. However, in some patients, vision loss progresses despite successful IOP control, indicating that new and effective treatments are needed, such as those targeting the neuroprotection of RGCs. Adenosine A_3_ receptor (A_3_R) activation confers protection to RGCs following an excitotoxic stimulus. In this work, we investigated whether the activation of A_3_R could also afford protection to RGCs in the laser-induced ocular hypertension (OHT) model, a well-characterized animal model of glaucoma. The intravitreal injection of 2-Cl-IB-MECA, a selective A_3_R agonist, abolished the alterations induced by OHT in the negative and positive components of scotopic threshold response (STR) without changing a- and b-wave amplitudes both in scotopic and photopic conditions. Moreover, the treatment of OHT eyes with the A_3_R agonist promoted the survival of RGCs, attenuated the impairment in retrograde axonal transport, and improved the structure of the optic nerve. Taking into consideration the beneficial effects afforded by 2-Cl-IB-MECA, we can envisage that A_3_R activation can be considered a good therapeutic strategy to protect RGCs from glaucomatous damage.

## Introduction

Glaucoma is a leading cause of blindness worldwide. It is estimated that in 2020, the global population with moderate or severe vision impairment by glaucoma will rise to 4.5 million, and the global population that go blind because of glaucoma will rise to 3.2 million^1^. This disease is characterized by optic nerve degeneration and loss of retinal ganglion cells (RGCs) that contribute to the vision loss^2,3^. The predominant and the only modifiable risk factor for the onset and progression of glaucoma is elevated intraocular pressure (IOP). Despite the growing research in the field of glaucoma, the current strategies only target lowering IOP either by topical administration of eye drops, laser, or incisional surgery. However, despite a 25% reduction in IOP in treated patients, half of them still progress in terms of visual deficits^4^. Moreover, normal tension glaucoma represents ~50% of glaucoma cases, and in the clinical practice, antihypertensive drugs remain the mainstay of treatment^5^. However, even in patients with controlled IOP the disease still progresses^6^. This demonstrates that IOP-independent mechanisms also contribute to the progression of the disease, suggesting that an effective therapeutic strategy should incorporate hypotensive drugs and neuroprotective agents, aiming at preserving RGCs^7^.

Adenosine is a neuromodulator acting through four adenosine receptors (A_1_, A_2A_, A_2B_, and A_3_)^8^. There is clinical and experimental evidence that activation of adenosine A_3_ receptor (A_3_R) mediates protective effects in ischemic brain injury^9^. Moreover, the activation of A_3_R protects RGCs against P2X7 receptor agonist-induced cell death^10,11^, and it limits the rise in intracellular calcium concentration evoked by stimulation of the NMDA receptor^12^. The protective effects may result from the direct action on RGCs, since these cells are endowed with A_3_R^13^. Moreover, we found that A_3_R activation prevents retinal cell death in several in vitro and animal models of retinal degeneration^14^, but the beneficial properties of 2-Cl-IB-MECA were not studied in animals with ocular hypertension (OHT). In this study, we investigated the therapeutic potential of 2-Cl-IB-MECA administered by intravitreal injection immediately after inducing OHT.

## Materials and methods

### Animals

Female Sprague–Dawley rats (Charles River, Spain) 8-weeks old were housed in animal facilities of the University of Murcia, Spain, in a 12 h light/12 h dark cycle, with free access to food and water. All procedures with animals were approved by the Ethical and Animal Studies Committee of the University of Murcia, and were in accordance with the Association for Research in Vision and Ophthalmology and European Union guidelines for animal research use.

### Induction of OHT and treatment with A3R agonist

Animals were anesthetized with an intraperitoneal injection of a mixture of ketamine (60 mg/kg, Ketalar, Pfizer, USA) and xylazine (10 mg/kg, Rompun, Bayer, Germany). OHT was induced in the left eyes in a single session with a series of diode laser burns (Viridis Ophthalmic Photocoagulator-532 nm, Quantel Medical, France), as previously described^15,16^. Immediately after OHT induction, both eyes (OHT and contralateral eyes) were treated with sterile saline solution (0.9% NaCl, 5 µl) or with 2-Cl-IB-MECA (1.2 µM, 5 µl) by intravitreal injection, the same dose used in our previous study^14^. Topical ointment with tobramycin (Tobrex, Alcon Cusí, S.A., Spain) was used to prevent corneal desiccation.

The animals were randomly assigned into naïve, saline-treated and 2-Cl-IB-MECA-treated group, and were sacrificed 7 days after OHT induction. IOP was monitored bilaterally 24 h after OHT induction. The IOP increased (35 ± 1.6 mmHg) in the left eyes, comparing with contralateral eyes (9 ± 0.2 mmHg).

### Electroretinography

At 6 days after OHT induction, the animals were dark-adapted overnight before the electroretinography (ERG) recordings. To carry out the recordings, we used a dim red light (*λ* > 600 nm) that allowed us to handle the equipment and the animals, while the animals remained in scotopic conditions. The rats were anaesthetized with an intraperitoneal injection of a mixture of ketamine (60 mg/kg, Ketalar, Pfizer, USA) and xylazine (10 mg/kg, Rompun, Bayer, Germany), and maintained on a heating pad to keep the body temperature. Pupil mydriasis was induced by applying a topical drop of 1% tropicamide (Colircusi tropicamida 1%®, Alcon Cusí, S.A., Spain) to both eyes, 5 min before ERG testing. Scotopic threshold responses (STR), and scotopic and photopic ERG responses were recorded in response to light stimuli produced by a Ganzfeld stimulator using Burian–Allen bipolar electrodes (Hansen Labs, USA) located on the cornea. The corneal surface had been previously protected with a nonallergenic ionic conductive drop of methylcellulose (methocel 2%, OmniVision, USA). The reference electrode was placed on the mouth, and the ground electrode was a needle placed subcutaneously at the base of the tail.

The STR was recorded by stimulating both eyes with −4.7 log cd·s/m^2^ of light intensity, and a series of ERG responses were averaged (~20 ERG responses) for each trace. The ERG responses were recorded by stimulating the retina with light intensities ranging between −1.69 to 2.19 log cd·s/m^2^ for scotopic a-wave, −3.61 to 2.19 log cd·s/m^2^ for scotopic b-wave, and 2.19 log cd·s/m^2^ for photopic b-wave. For each light intensity, a series of ERG responses were averaged (~40 ERG responses for the dimmest stimulus intensities to 5 ERG responses for the brightest stimulus) with an interval between light flashes from 5 s for the dimmest stimulus intensities to 60 s for the brightest stimulus.

Electrical signals were digitized at 20 kHz using a Power Lab data acquisition board (AD Instruments, Australia) and displayed on a PC computer. The light stimuli were calibrated with a photometer (Mavo Monitor USB, Gossen, Germany). The STR was analyzed for each stimulus as follows: positive STR (pSTR) was measured from the baseline to the peak of the positive deflection, ~110–120 ms from the flash onset; negative STR (nSTR) was measured from the baseline to the peak of the negative deflection after the pSTR, ~220 ms from the onset of the flash. The investigator was blinded to the group when performing the experiment and extracting data. Standard ERG waves were analyzed according to the method recommended by the International Society for Clinical Electrophysiology of Vision^17,18^.

### Retrograde tracing of RGCs

After 24 h of laser-induced OHT procedure, animals were anesthetized using the aforementioned anesthetic protocol. Fluorogold (FG, Fluorochrome Inc., USA) was prepared at 3% concentration (w/v) in a solution of 10% DMSO-saline, and it was applied onto the surface of both superior colliculi (SCi). The animals were sacrificed 6 days after FG application (7 days after OHT induction), and the retinas were processed for whole mount preparation.

### Immunolabelling

#### In retinal whole mounts

Animals were euthanized with an intraperitoneal injection overdose of pentobarbital (Dolethal, Vetoquinol, France), and perfused with PBS followed by 4% paraformaldehyde (PFA). Then, eyecups were enucleated and fixed for an additional hour in 4% PFA. For retinal whole mounts, the eyes were maintained in PBS until dissection as flat mounts. Retinal whole mounts were permeabilized with 0.5% Triton X-100 and incubated with primary antibody (goat anti-Brn3a, catalog number sc-31984, Santa Cruz Biotechnology, USA) overnight at 4 °C. Retinas were incubated with the secondary antibody (donkey anti-goat IgG conjugated to Alexa Fluor 594, catalog number A11058, Thermo Fisher Scientific, USA), and mounted with the vitreous side up and covered with anti-fading mounting medium.

#### In retinal cryosections

Immunohistochemistry in retinal cryosections was performed as previously described^19^. The sections were incubated overnight with the primary antibodies: rabbit anti-A_3_R (catalog number sc-13938, Santa Cruz Biotechnology; USA), mouse anti-Brn3a (catalog number MAB1585, Millipore, USA), and rabbit anti-RBPMS (RNA-binding protein with multiple splicing; catalog number ab194213, Abcam, United Kingdom). The sections were rinsed with PBS followed by incubation with the corresponding secondary antibodies for 1 h at room temperature in the dark: goat anti-rabbit conjugated to Alexa Fluor 488 (catalog number A11008, Thermo Fisher Scientific, USA), goat anti-mouse conjugated to Alexa Fluor 568 (catalog number A11004, Thermo Fisher Scientific, USA), and goat anti-rabbit conjugated to Alexa Fluor 568 (catalog number A11036, Thermo Fisher Scientific, USA). For the counting of RBPMS^+^ cells, the preparations were observed in a fluorescence microscope (Axio Observer.Z1, Zeiss, Germany), using a 20× objective (Plan Achromat 20×/0.8 M27). From each eye, four sections were analyzed and the number of RBPMS^+^ cells was counted in the entire retinal section and normalized to the length of the respective section. The RBPMS survival rate was presented as the percentage of the ratio between the OHT-injured retina and the contralateral eye. For both RBPMS and A_3_R immunohistochemistry, representative images were acquired with a 40× objective (EC Plan-Neofluar 40×/1.30 Oil DIC M27) on a confocal microscope (Zeiss LSM 710, Germany).

### Acquisition of FG and Brn3a labeling in retinal whole mounts

Whole-mounted retinas were acquired with a 10× objective in an epifluorescence microscope (Axioskop 2 Plus; Zeiss, Germany) equipped with a computer-driven motorized stage (ProScan H128 Series; Prior Scientific Instruments Ltd, United Kingdom), controlled by Image-Pro Plus (IPP 5.1 for Windows; Media Cybernetics, USA), as previously described^20^. FG^+^RGC and Brn3a^+^RGCs were automatically quantified as reported^20^. Reconstructed whole mounts, made up from 185 individual frames, were further processed for representative images using Adobe Photoshop® CS 8.0.1 (Adobe Systems, Inc., USA). The Brn3a survival rate was presented as the percentage of the ratio between the OHT-injured retina and the contralateral eye.

### Terminal deoxynucleotidyl transferase-mediated dUTP nick-end labeling assay

Cell death was detected in retinal cryosections with a terminal deoxynucleotidyl transferase-mediated dUTP nick-end labeling (TUNEL) assay kit, as we previously described^19^. The preparations were visualized in a fluorescence microscope (Axio Observer.Z1, Zeiss, Germany) with a 20× objective (Plan Achromat 20×/0.8 M27). From each eye, four sections were analyzed and the total number of TUNEL^+^ cells was counted in the entire retinal section and normalized to the length of the respective section.

### Real-time qPCR

Total RNA was extracted from rat retinas using Trizol reagent (Invitrogen, Thermo Fisher Scientific, USA), as we previously described^19^. SYBR Green-based qPCR was performed using StepOnePlus (Applied Biosystems, USA), with the following primers for Adora3 (F: GCTTGGATTACATGGTCTTC; R: TGAGTTTGTTTCGGATGATG) and *Hprt1* (F: ATGGGAGGCCATCACATTGT; R: ATGTAATCCAGCAGGTCAGCAA) was the most stable gene tested, and it was used as the control gene. Ct values were converted to ‘relative quantification’ using the 2^−ΔΔCt^ method described previously^21^.

### Transmission electron microscopy

Following transcardial perfusion, the brain was removed, and optic nerve samples were collected close to the optic chiasm. Samples were fixed with 2.5% glutaraldehyde in 0.1 M sodium cacodylate buffer (pH 7.2) for 2 h. Following three washing steps in buffer, postfixation was performed using 1% osmium tetroxide for 90 min. Samples were then rinsed in buffer, dehydrated in a graded ethanol series (70–100%), and embedded in 2% molten agar. Sample pellets were redehydrated in ethanol (30–100%) and then, impregnated and included in Epoxy resin (Sigma-Aldrich, USA). Ultrathin sections were mounted on copper grids and observations were carried out on a FEI Tecnai G2 Spirit BioTWIN (FEI Company, USA) at 100 kV.

### Statistical analysis

The results are presented as mean ± standard error of the mean. Data points were excluded if identified as outliers with the ROUT algorithm using Prism (GraphPad Software). Statistical analysis was performed with the Prism 5.03 Software for Windows (GraphPad Software, Inc, USA). The normality of the data was assessed with Shapiro–Wilk normality test, and data were analyzed with parametric or nonparametric tests, depending on data distribution.

## Results

In order to assess the protective effects of the selective A_3_R agonist, 2-Cl-IB-MECA was administered by intravitreal injection (1.2 µM, 5 µl) immediately after the induction of OHT.

### Distribution of A3R in the retina

**Fig. 1:**
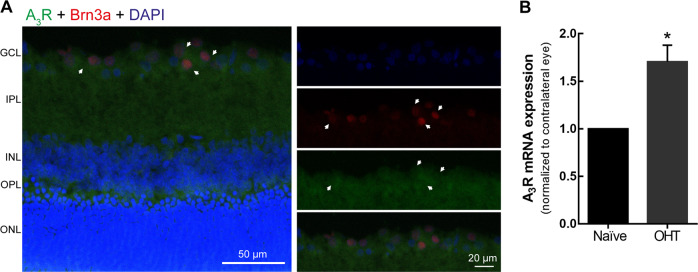
A_3_R is upregulated after 7 days of OHT induction. **a** Representative images of naïve retinal cryosections immunostained for A_3_R (green) and Brn3a (RGCs, red) are depicted. Nuclei were stained with DAPI (blue). **b** A_3_R mRNA expression was assessed by qPCR in naïve and OHT retinas. Results are presented as fold change of OHT eye to naïve, from seven independent experiments. *p < 0.05, significantly different from the naïve, Wilcoxon’s signed-rank test. GCL ganglion cell layer, IPL inner plexiform layer, INL inner nuclear layer, OPL outer plexiform layer, ONL outer nuclear layer.

Previous studies have identified messenger RNA (mRNA) coding for A_3_R in rat RGCs^13^. In retinal vertical sections from naïve animals, the immunoreactivity of A_3_R was mainly observed in the GCL in Brn3a^+^ cells (Fig. 1a), confirming that RGCs are endowed with A_3_R. The effect of OHT on the levels of A_3_R mRNA in the retina was determined by qPCR. OHT caused an increase of 1.7-fold in A_3_R mRNA expression in the retina (Fig. 1b).

### Treatment with 2-Cl-IB-MECA attenuates the RGC dysfunction induced by OHT

**Fig. 2:**
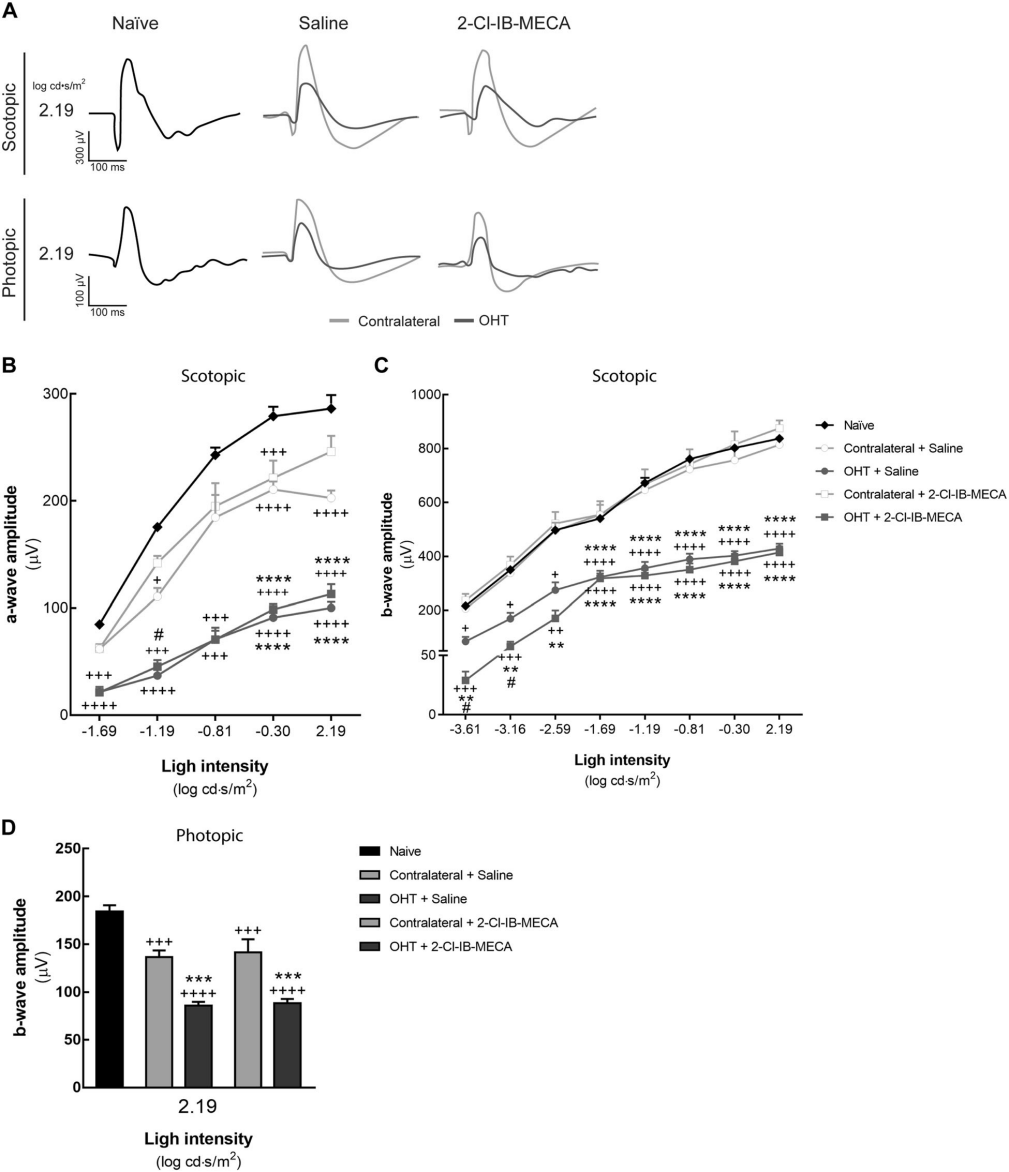
Treatment with 2-Cl-IB-MECA does not change a-wave and b-wave amplitudes. Saline (5 µl) or 2-Cl-IB-MECA (5 µl, 1.2 µM) was administered by intravitreal injection immediately after laser-induced OHT, and at day 6 after OHT induction the ERG was recorded. **a** Representative traces of scotopic a- and b-wave amplitude and photopic b-wave at 2.19 log cd·s/m^2^. **b**, **c** Scotopic a- and b-wave amplitudes recorded at different light intensities. **d** Photopic b-wave amplitudes recorded at 2.19 log cd·s/m^2^. Results presented were obtained from 5–6 animals. ^+^*p* < 0.05, ^++^*p* < 0.01, ^+++^*p* < 0.001, ^++++^*p* < 0.0001, significantly different from naïve; *p < 0.05, **p < 0.01, ***p < 0.001, ****p < 0.0001, significantly different from contralateral eye; and ^#^*p* < 0.05, ^##^*p* < 0.01, significantly different from saline-treated OHT.

Retinal function was assessed by ERG, a record of electrical responses in the eye obtained by stimulating the retina with light flashes in either dark-adapted (scotopic) or light-adapted (photopic) conditions (Fig. 2a). The a-wave is the first major component of ERG that corresponds to the function of photoreceptors, being in scotopic conditions mainly due to mixed rod and cone response, and in photopic conditions mainly due to cones response^22^. Evidence suggests that the b-wave originates in retinal cells that are postsynaptic to photoreceptors^22^.

Generally, when retinal function deteriorates, the light-induced electrical activity in the retina reduces^23^. As expected, the amplitude for scotopic a- and b-waves increased with the increase of light intensity in naïve animals (Fig. 2b, c). In contralateral retinas, the amplitude of a-wave in scotopic conditions (Fig. 2b) and of b-wave in photopic conditions (Fig. 2d) decreased when compared with naïve retinas, independently if treated with saline or 2-Cl-IB-MECA. In OHT retinas, the amplitudes of scotopic a- and b-waves, as well as the amplitude of photopic b-wave decreased. These effects were not modified by 2-Cl-IB-MECA, except at lower light flash stimuli in scotopic b-wave.

**Fig. 3:**
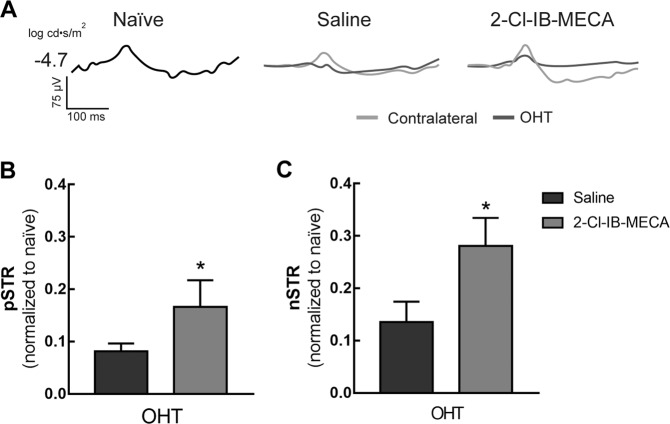
Treatment with 2-Cl-IB-MECA attenuates the loss-of-function of RGCs induced by OHT. Saline (5 µl) or 2-Cl-IB-MECA (5 µl, 1.2 µM) was administered by intravitreal injection immediately after laser-induced OHT, and at day 6 after OHT induction the STR was recorded. **a** Representative traces of STR recordings. **b**, **c** pSTR and nSTR amplitudes recorded at −4.7 log cd·s/m^2^, extracted from 5–6 animals. *p < 0.05, significantly different from the saline-treated OHT.

The function of RGCs can be assessed by ERG using very dim light intensities after extracting the positive and negative components^24^. In this work, STR was elicited with light stimuli of −4.7 log cd·s/m^2^ (Fig. 3a), and the amplitudes of each positive (pSTR, Fig. 3b) and negative (nSTR, Fig. 3c) components were extracted. OHT decreased the amplitudes of both pSTR and nSTR, and the treatment with 2-Cl-IB-MECA was able to reduce the effect of OHT in pSTR and nSTR amplitudes (*p* < 0.05).

### A_3_R receptor agonist does not change retinal cell death induced by OHT

**Fig. 4:**
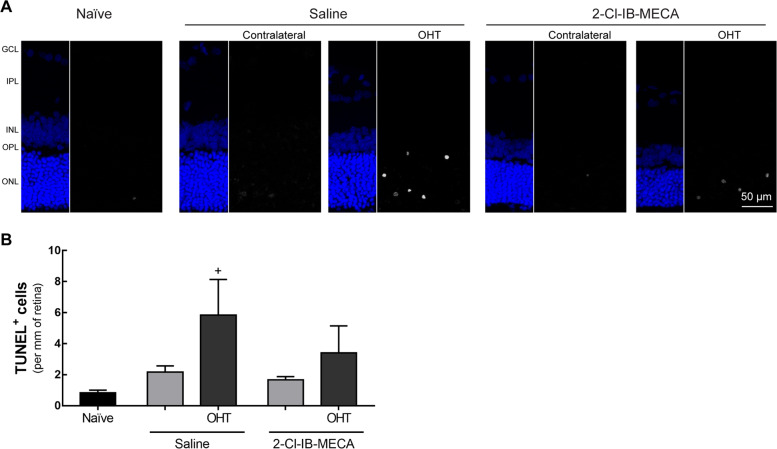
Treatment with 2-Cl-IB-MECA reduces retinal cell death induced by OHT. Saline (5 µl) or 2-Cl-IB-MECA (5 µl, 1.2 µM) was administered by intravitreal injection immediately after laser-induced OHT. **a** Cell death was assayed in retinal cryosections by TUNEL assay (white). Nuclei were stained with DAPI (blue). Representative images are depicted. **b** The total number of TUNEL^+^ cells were counted in the entire retinal section and expressed per length (mm) of the respective retinal section from 5–6 independent experiments. ^+^*p* < 0.05, significantly different from naïve. GCL ganglion cell layer, IPL inner plexiform layer, INL inner nuclear layer, OPL outer plexiform layer, ONL outer nuclear layer.

The TUNEL assay was performed in retinal vertical sections to quantify cell death (Fig. 4). The majority of TUNEL^+^ cells was found in the outer nuclear layer (ONL) and INL (Fig. 4a), indicating that cells in these layers are also affected by OHT, in accordance with a previous report^25^. No significant changes in TUNEL^+^ cells were found in the retinas of contralateral eyes for both groups of animals (saline-treated and 2-Cl-IB-MECA-treated group), when compared with naïve animals. In the OHT-injured retinas treated with saline there were 5.8 ± 2.3 TUNEL^+^ cells/mm (*p* < 0.05, compared with naïve) and in the 2-Cl-IB-MECA-treated retinas there were 3.4 ± 1.7 TUNEL^+^ cells/mm (Fig. 4b).

### Activation of A3R receptor increases the survival of RGCs in animals with OHT

**Fig. 5:**
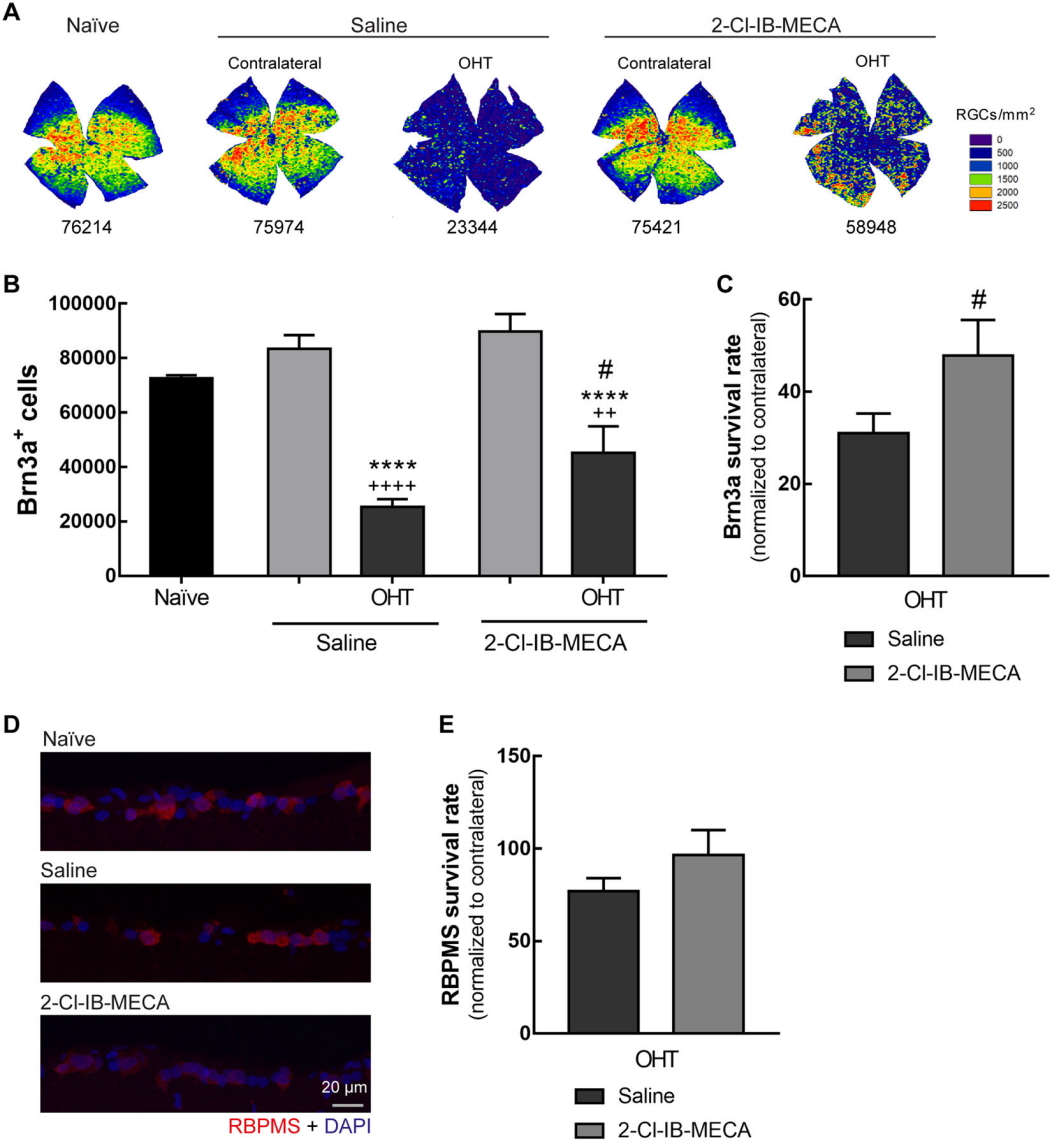
A_3_R agonist increases RGC survival in OHT animals. Saline (5 µl) or 2-Cl-IB-MECA (5 µl, 1.2 µM) was administered by intravitreal injection immediately after laser-induced OHT. **a** Representative RGC isodensity maps generated from whole-mount preparations from 2-Cl-IB-MECA and saline-treated OHT, and contralateral retinas immunostained for Brn3a. **b** The number of total Brn3a^+^ cells per retina was automatically quantified from 5–6 independent retinas. **c** The survival rate of Brn3a^+^ cells was expressed as the percentage of the ratio between OHT-injured retinas and the contralateral retinas, from 5–6 independent experiments. **d** Representative images of retinal cryosections immunostained for RBPMS (red). Nuclei were stained with DAPI (blue). **e** The number of RBPMS^+^ cells were counted in the entire retinal section and normalized to the length of the respective section. The survival rate of RBPMS^+^ cells was presented as the percentage of the ratio between the OHT-injured retinas and the contralateral retinas, from 5–6 independent experiments. ^++^*p* < 0.01, ^++++^*p* < 0.0001, significantly different from naïve; ****p < 0.0001, significantly different from the contralateral eye; and ^#^*p* < 0.05, significantly different from saline-treated OHT.

Since RGCs express A_3_R^13^ and 2-Cl-IB-MECA attenuated the effect of OHT in STR, we assessed whether the treatment with the A_3_R agonist could protect RGCs. In retinal whole mounts, RGCs were immunolabelled for Brn3a, a specific marker of RGCs^26^, and the isodensity maps allowed us to visualize the distribution of RGCs (Fig. 5a). The distribution of Brn3a^+^ RGCs in both naïve and contralateral retinas is similar to a previous report^26^, with higher density in the superior retina and a visual-oriented horizontal strip. The total number of Brn3a^+^ cells was automatically counted (Fig. 5b). In naïve retinas, the number of Brn3a^+^ cells was 72,501 ± 1237 cells, similar to our previous work^16^. The treatment of contralateral retinas with saline or 2-Cl-IB-MECA did not significantly affect the number of Brn3a^+^ cells (83,406 ± 5067 cells and 89,689 ± 6517 cells, respectively) when compared with naïve retinas. As expected, laser-induced OHT induced a significant loss of Brn3a^+^ cells (25,328 ± 2862 cells, *p* < 0.01) that was partially attenuated by the intravitreal injection of 2-Cl-IB-MECA (45299 ± 9640 cells, *p* < 0.05; Fig. 5b). In fact, the animals treated with 2-Cl-IB-MECA presented 48% of Brn3a^+^ cells, which is significantly (*p* < 0.05) higher when comparing with saline-treated group (survival rate of 31%; Fig. 5c).

RBPMS is another RGC marker for quantitative analysis of these cells in animal models of RGC degeneration induced by IOP elevation^27,28^. Therefore, the number of RBPMS^+^ cells was counted in retinal cryosections (Fig. 5d). Although not so pronounced, 2-Cl-IB-MECA slightly increased the number of RBPMS^+^ cells present in OHT retinas (survival rate of 96%) comparing with saline-treated group (survival rate of 77%; Fig. 5e).

### A_3_R receptor agonist prevents structural alterations in the optic nerve induced by OHT and ameliorates the OHT-induced impairment in the optic nerve retrograde transport

**Fig. 6:**
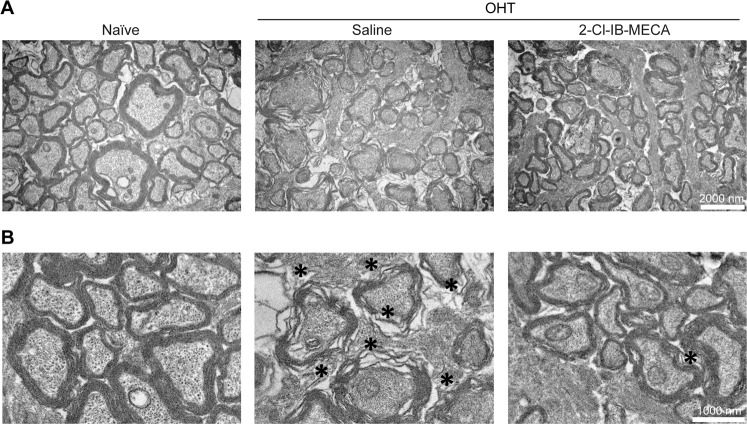
Treatment with 2-Cl-IB-MECA prevents the structural alterations of optic nerve induced by OHT. Saline (5 µl) or 2-Cl-IB-MECA (5 µl, 1.2 µM) was administered by intravitreal injection immediately after laser-induced OHT. Semi-thin cross sections of naïve, saline-, and 2-Cl-IB-MECA-treated OHT retinas were imaged by transmission electron microscopy. Representative images are depicted at (**a**) low magnification (scale bar: 2000 nm) and (**b**) higher magnification (scale bar: 1000 nm). Structural alterations like degenerating axons and myelin disarrangement are observed in OHT animals (indicated by black asterisks).

Changes in the structure of the optic nerves were assessed by transmission electron microscopy (Fig. 6). The optic nerves from animals with OHT presented regions with disorganized axons, including alterations in the myelin sheath (Fig. 6b, indicated by black asterisks). The treatment with 2-Cl-IB-MECA was able to partially halt the alterations caused by OHT.

**Fig. 7:**
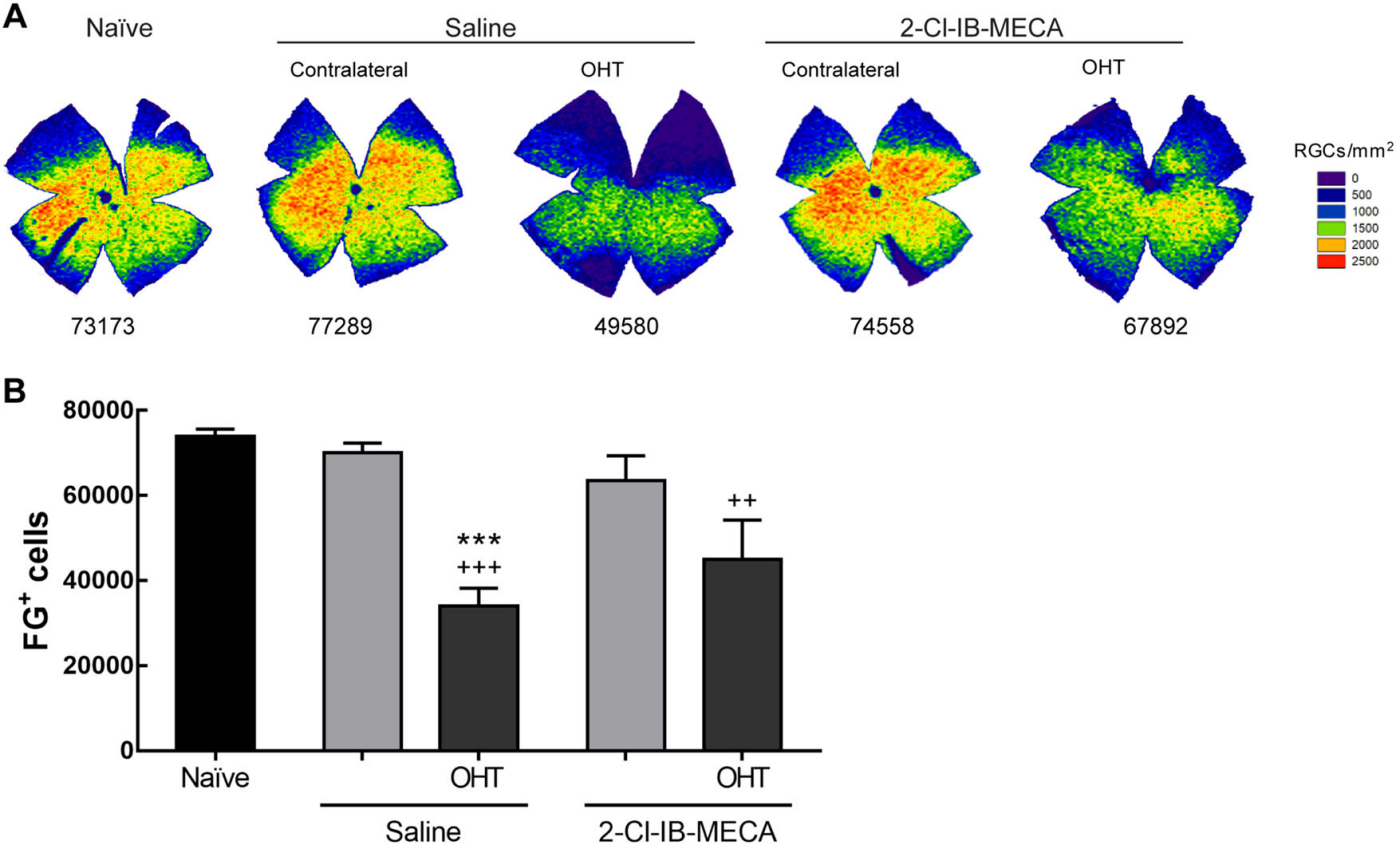
Treatment with 2-Cl-IB-MECA attenuates the impairment in axonal transport induced by OHT. Saline (5 µl) or 2-Cl-IB-MECA (5 µl, 1.2 µM) was administered by intravitreal injection immediately after laser-induced OHT. **a** Retrograde axonal transport was assessed after FG application in the superior colliculus (SCi) at 24 h after OHT induction. Representative isodensity maps generated from whole-mount preparations from 2-Cl-IB-MECA, and saline-treated OHT and contralateral retinas labeled with FG. **b** The total number of FG^+^ cells was automatically quantified from 5–6 independent experiments. ^++^*p* < 0.01, ^+++^*p* < 0.001 significantly different from naïve; and ***p < 0.001 significantly different from contralateral eye.

One feature of laser-induced OHT is the impairment of the axonal transport through the optic nerve^15^. Therefore, since A_3_R agonist was able to protect RGCs, we assessed if the treatment with 2-Cl-IB-MECA could change the course of the disease. Optic nerve retrograde transport was assessed by counting the number of FG^+^ cells in the retina after application of the dye in the SCi (Fig. 7). The induction of OHT did not significantly change the number of FG^+^ cells in the contralateral retinas (70,067 ± 2271 cells and 63,504 ± 5832 cells, for saline or 2-Cl-IB-MECA, respectively) when compared with naïve retinas (73,949 ± 1653 FG^+^ cells); also similar to our previous report^16^. The number of FG^+^ cells significantly decreased to 34,122 ± 4090 cells in OHT retinas, but the treatment with 2-Cl-IB-MECA was able to attenuate the effect of OHT (44,934 ± 9301 FG^+^ cells; Fig. 7b). This result suggests that A_3_R agonist might improve the axonal transport through the optic nerve.

## Discussion

The results presented herein demonstrate that the treatment with an agonist of A_3_R confers protection to RGCs against damage induced by OHT. In addition, we showed that the activation of A_3_R attenuated the loss of function of RGCs and the alterations of their axons. Previously, we demonstrated that the activation of A_3_R protects the retina, including RGCs against transient ischemic damage^14^. However, in this previous work, the A_3_R agonist 2-Cl-IB-MECA was administered prior injury and the protective effects were evaluated 24 h after. In the current work, we extended the previous knowledge by evaluating the potential therapeutic properties of A_3_R agonist in a model of OHT by administering the drug immediately after inducing OHT, and assessing the outcome 7 days after the treatment. Previously, we showed that the A_3_R-selective antagonist (MRS 1191) abolishes the protective effects of the A_3_R agonist against cell death induced by glutamatergic excitotoxicity in primary rat retinal neural cultures and in retinal organotypic cultures^14^, showing that the protective effects are mediated by A_3_R activation.

Several animal models have been developed to mimic glaucoma and the laser photocauterization of the perilimbar and episcleral veins to induce OHT has been a widely used method in adult albino rats^23^. In this animal model, IOP raises in the first 24 h post OHT induction and it is constantly maintained elevated during the first week^25^. Our study was conducted to assess the protective properties of 2-Cl-IB-MECA against the loss of RGCs induced by OHT within the period of elevated IOP to avoid possible confounding effects of IOP normalization.

Apoptotic cell death has been described in glaucomatous patients and experimental models^29–31^. In the OHT animals, the majority of apoptotic cells (TUNEL^+^ cells) were located at the ONL. It has been proposed that glaucoma causes not only RGC death, but also degeneration of cells in the ONL, most likely photoreceptors that are lost due to laser-induced photocoagulation^17,25,32,33^. In fact, death of photoreceptors has also been described in glaucoma patients^34–36^. In the animals with OHT, the decrease in the amplitudes of a- and b-waves of the full-field ERG in scotopic and photopic conditions strongly suggests that other cells apart from RGCs are affected in glaucomatous conditions. Surprisingly, TUNEL^+^ cells were not observed in the GCL, despite the loss of RGCs. Retinal microglia are the resident immune cells that become reactive in the retinas of laser-induced OHT eyes^37^. One possible explanation for the lack of TUNEL^+^ cells in GCL could be the fact that microglial cells are actively clearing the tissue from dead RGCs. In fact, we recently demonstrated that elevated pressure increases the number of engulfed TUNEL^+^ cells by microglia^38^ and the observation of transcellularly labeled microglial cells with FG also favors this hypothesis^39^.

Beyond RGC dysfunction, axonal transport is also impaired in experimental glaucoma^15,40,41^. FG has been the tracer of choice to evaluate retrograde transport through axons of RGCs from SCi to the retina^24^. The tracer can be applied in SCi and the number of FG^+^ cells in the retina can be easily counted^26^. In the current study, FG was administered after laser photocoagulation in order to guarantee that FG^+^ cells represent the RGCs with non-impaired axonal transport. In the laser-induced OHT group, impaired retrograde axonal transport and RGC loss were found, and are in accordance with previous reports^15,25^.

We found that OHT impacted the amplitudes of scotopic a-wave and photopic b-wave in the contralateral retinas. These alterations were never reported previously and reinforce the importance of appropriate controls (naïve and contralateral) when assessing the changes triggered by OHT. In fact, bilateral response to experimental injury in rodents has been described to cause, at least, microglial activation^42,43^ and RGC loss^44,45^.

A_3_R has been implicated in many ocular diseases, like autoimmune uveitis, dry eye syndrome, and glaucoma^46^. The safety and efficacy of CF101 (IB-MECA, an agonist for A_3_R) is being assessed in a randomized clinical trial in patients with elevated IOP (NCT01033422). CF101 was able to decrease IOP in a dry eye syndrome phase II clinical study^47^. In fact, A_3_R contributes to the regulation of IOP^48^, and data from the clinical trial demonstrate that CF101 was effective as an IOP-lowering agent^47^. A_3_R was identified in rat RGCs^13^, and more recently also in nerve fiber layer and retinal pigment epithelium of Rhesus monkeys^49^. There are no evidences of A_3_R expression in the outer retina of rodents. However, it has been suggested that A_3_R may be located in neurons of the inner retina that would contribute to the generation of the ERG a- and b-waves^50^. Overall, in our experimental conditions, no changes were observed in a- and b-waves amplitudes in scotopic and photopic ERG, but different drug concentration and timepoints may help explaining these differences.

The activation of A_3_R attenuated the decrease in FG^+^ cells and increased the survival of Brn3a^+^ cells induced by OHT. The degenerative process of RGCs is accompanied by structural alterations in RGC axons^51^. The intravitreal injection of 2-Cl-IB-MECA preserved the structure of the optic nerve, consistent with the data on FG axonal transport. The A_3_R agonist 2-Cl-IB-MECA promotes neurite outgrowth in cultured RGCs and axonal regeneration in the optic nerve crush model through the activation of an Akt-dependent signaling pathway^52^. In our model, similar pathways could be activated in the RGC soma, which may contribute to the improvement in axonal transport.

It is well established that RGCs are lost in the laser-induced OHT animal model of glaucoma^15,25^. Interestingly, even using different markers (Brn3a and RBPMS) and preparations for assessing RGC loss (retinal cryosections and whole mounts), the magnitude found for the protection of RGCs due to the treatment with the A_3_R agonist was very similar. The A_3_R agonist increases the survival of Brn3a^+^ RGCs by 54% (survival rate: 31% in saline-treated group and 48% in 2-Cl-IB-MECA-treated group) and by 24% in the case of RBPMS^+^RGCs (survival rate: 77% in saline-treated group and 96% in 2-Cl-IB-MECA-treated group). There is some controversy regarding the most reliable marker for RGC, especially in the case of RGC degeneration^24^. Brn3a is a POU-domain transcription factor expressed in RGCs^53^ and is downregulated in injured RGCs^54^ in a caspase-3-dependent pathway^55^. However, since this downregulation occurs near the death of RGC, it does not hinder accurate counting of RGCs using Brn3a, as a cell marker^26,56^. Nevertheless, some authors report a downregulation of Brn3a prior RGCs loss^24^, suggesting the use of other markers. RBPMS has been proposed as a marker of RGCs even in the case of neurodegeneration^27,28^. Despite the different extension of the injury when comparing Brn3a^+^RGCs and RBPMS^+^RGCs, the protection conferred by 2-Cl-IB-MECA is similar comparing both markers. Therefore, regardless the most reliable marker for RGCs, there is no doubt of the protective properties of 2-C-IB-MECA. The STR is considered to reflect the activity of RGCs^24^. Indeed, a single intravitreal injection of A_3_R agonist was able to attenuate the effect of OHT on the amplitude of pSTR and nSTR elicited at −4.7 log cd·s/m^2^. The mechanism by which A_3_R activation mediates protection to RGCs may involve a decrease in calcium influx^12^, although this was not addressed in this work. Indeed, it has been described that an increase in calcium influx in RGCs mediated by transient receptor potential vanilloid 1 channel activation leads to pressure-induced RGCs death^57^.

One important consideration is that the effects mediated by A_3_R agonist were only assessed 7 days after one single injection. Although repeated injections may cause several side effects, such as inflammation, endophthalmitis, retinal detachment, and cataracts^58^, one could hypothesize that the beneficial effects of A_3_R activation could be potentiated if other therapeutic regimens had been adopted.

Taking together, these results demonstrate that A_3_R activation may be a promising novel therapeutic strategy focusing on the protection of RGCs for the treatment of glaucoma.

## Conclusions

Glaucoma is characterized by the loss of RGCs and degeneration of their axons, affecting cell function. The current treatments for this disease are dependent on the control of IOP, the only modifiable risk factor. However, in some patients despite having controlled IOP, the disease still progresses. Therefore, there is an emergent need for new therapeutic strategies to manage glaucoma. Drugs targeting RGCs protection may have potential to be effective for the treatment of glaucoma, additionally to IOP-lowering agents. The treatment with A_3_R agonist prevented the loss of RGCs, and attenuated the loss-of-function of RGCs and the retrograde axonal transport failure induced by OHT. Concluding, our data shed light on a novel potential therapeutic strategy for glaucoma, using the A_3_R activation as an IOP-independent neuroprotective therapeutic strategy for glaucoma.
